# Regulation of Autophagy by Glucose in Mammalian Cells

**DOI:** 10.3390/cells1030372

**Published:** 2012-07-27

**Authors:** Félix Moruno, Eva Pérez-Jiménez, Erwin Knecht

**Affiliations:** 1 Principe Felipe Research Centre, C/Eduardo Primo Yúfera 3, Valencia 46012, Spain; Email: jomoman@upvnet.upv.es; 2 Center for Biomedical Research on Rare Diseases (CIBERER), Valencia 46012, Spain

**Keywords:** autophagy, regulation, nutrients, energy, glucose, AMPK

## Abstract

Autophagy is an evolutionarily conserved process that contributes to maintain cell homeostasis. Although it is strongly regulated by many extracellular factors, induction of autophagy is mainly produced by starvation of nutrients. In mammalian cells, the regulation of autophagy by amino acids, and also by the hormone insulin, has been extensively investigated, but knowledge about the effects of other autophagy regulators, including another nutrient, glucose, is more limited. Here we will focus on the signalling pathways by which environmental glucose directly, *i.e.*, independently of insulin and glucagon, regulates autophagy in mammalian cells, but we will also briefly mention some data in yeast. Although glucose deprivation mainly induces autophagy via AMPK activation and the subsequent inhibition of mTORC1, we will also comment other signalling pathways, as well as evidences indicating that, under certain conditions, autophagy can be activated by glucose. A better understanding on how glucose regulates autophagy not only will expand our basic knowledge of this important cell process, but it will be also relevant to understand common human disorders, such as cancer and diabetes, in which glucose levels play an important role.

## Abbreviations

2-DG: 2-deoxy-D-glucose
AICAR: 5-aminoimidazole-4-carboxamide-1-β-D-ribofuranoside
AMPK: AMP-activated protein kinase
ATG: autophagy-related
ATP: adenosine triphosphate
ER: endoplasmic reticulum
ERK: extracellular signal-regulated protein kinase
FOXO: forkhead class O
IKK: IκB kinase
JNK: c-Jun amino-terminal kinase
PAS: preautophagosomal structure
PI3K: phospahtidylinositol 3-kinase
PKA: cAMP-dependent protein kinase A
ROS: reactive oxygen species
TAB: TAK-binding protein
V-ATPase: vacuolar ATPase

## 1. Introduction

**Figure 1:**
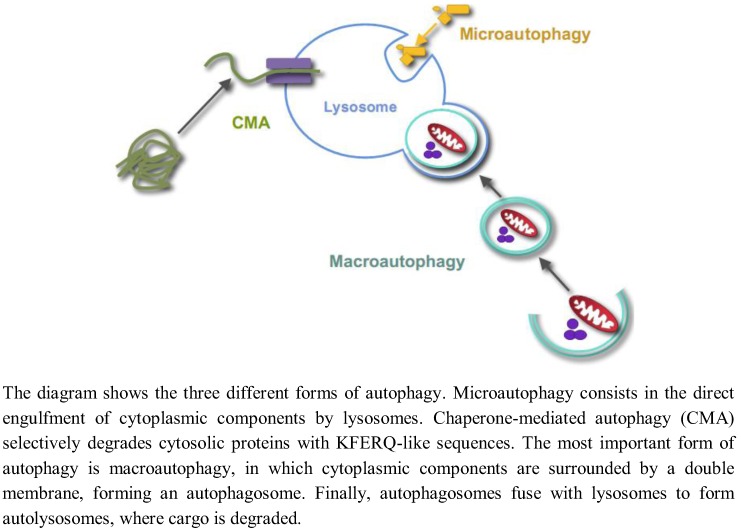
Different forms of autophagy.

Autophagy is the process by which lysosomes degrade organelles and cellular components. Three different forms of autophagy coexist in most cells (Figure 1): Microautophagy, chaperone-mediated autophagy and macroautophagy [1].

Microautophagy, a still poorly characterized type of autophagy in mammalian cells, consists in the direct engulfment of cytoplasmic components in intralysosomal tubules and vesicles, which are produced by various modifications of the lysosomal membranes. Chaperone-mediated autophagy is a selective lysosomal pathway for the degradation of cytosolic proteins with KFERQ-like sequences, in which both cytosolic and lysosomal chaperones facilitate the transport of these proteins for degradation into the lysosomal lumen through a multimer of the lysosomal protein LAMP2A spanning the lysosomal membrane. Macroautophagy is the most important and better known form of autophagy. Initially, cytoplasmic components are surrounded by a double membrane, with still an unclear origin, forming a vacuole, which is called an autophagosome. Autophagosomes fuse with endosomes and lysosomes to form autolysosomes with an acidic pH and where the degradation of the sequestered material by lysosomal hydrolases occurs. While autophagy has been usually considered a nonselective and an in bulk degradation process, there are also selective autophagic pathways. For example, the selective degradation of mitochondria, peroxisomes or ribosomes by autophagy is called, respectively, mitophagy, pexophagy or ribophagy. In this paper, unless otherwise stated, the term “autophagy” will only refer to macroautophagy, and we will not review here the two other forms of autophagy.

Autophagy is regulated by a high number of extracellular stimuli, which are transduced through a large variety of signalling pathways with the kinase mTORC1 occupying a central position in most of them [2,3]. In mammalian cells, the main regulators of autophagy are hormonal and nutritional factors. Among those, the regulation by amino acids and insulin, their signalling pathways and the underlying mechanisms on the autophagic machinery have been investigated in most detail (see *e.g.* [4]). In contrast, the information on other molecules that also regulate autophagy is much scarce.

Autophagy, like protein degradation [5], in spite of being a catabolic process requires an input of energy, as first established by Plomp *et al.* [6]. ATP (adenosine triphosphate) is required for, at least, two autophagic steps: (i) sequestration of the cytosolic material in autophagosomes; and (ii) maintenance of the activity of the proton pump (vacuolar-type ATPase or V-ATPase) at the lysosomal membrane to produce the lysosomal acidification required in the autophagic flux. Since most ATP in the cell derives from glucose metabolism, this metabolite should be important for the normal functioning of autophagy. However, glucose is also a nutrient, and it is well known that nutrient depletion induces autophagy to provide the cell with energy and building blocks. Therefore, whether glucose affects positively or negatively autophagy is a matter of discussion, as we will see below. In this review, we aim to present an overview of what is specifically known on the signalling pathways that regulate autophagy in response to glucose or glucose-deprivation. Due to space limitations, we will mention only those papers dealing, more or less directly, with this question, while other specific stimuli regulating autophagy, other signalling pathways and the autophagy machinery will not be reviewed here. We will mainly concentrate on mammalian cells, but first we will give in the next section a brief and general account on this subject in yeast.

## 2. Regulation of Autophagy by Glucose in Yeast

Most of our current knowledge on the molecular machinery of autophagy derives from studies in yeast that, initially, were mainly carried out in the laboratories of Yoshinori Ohsumi in Japan, Michael Thumm in Germany and Dan Klionsky in USA. In the late 90s, two different forms of autophagy were recognized in yeast: micro- and macroautophagy [7]. The first was described in yeast as a process by which cell components are surrounded by cytoplasmic invaginations or finger-like protrusions of the vacuole, which eventually form intravacuolar vesicles that, together with their content, are degraded by the vacuolar hydrolytic enzymes. In macroautophagy, cytoplasmic components are surrounded by a membrane from a compartment called the preautophagosomal structure (PAS) that closes to form a double-membrane autophagosome [8]. Then, the outer membrane of the autophagosome fuses with the vacuolar membrane and the single membrane autophagic body that has entered into the vacuole is degraded by vacuolar hydrolases [9]. The identification of the *ATG* (autophagy-related) genes provided insights into the molecular basis of autophagy. Many of the codified proteins in yeast are conserved in mammals, but there are also mammalian specific ATG proteins. Now, the role of some, but not all, of the more than 30 identified Atg proteins is known and has been discussed elsewhere [10,11,12]. Most of them participate in the initiation of autophagy, but some others are essential for other autophagic steps.

In yeast, autophagy is mainly affected by nitrogen starvation, but glucose starvation plays also an important role, as we will see below in a few examples. The growth of *Saccharomyces cerevisiae* in a medium without glucose induces the synthesis of gluconeogenic enzymes (fructose-1,6-bis-phosphatase, malate dehydrogenase, phosphoenolpyruvate carboxykinase) and also of the galactose and maltose transporters. When these cells are subsequently incubated in a medium containing glucose, the transcription of these genes is suppressed and the induced proteins are degraded [13] in the general process of catabolite repression. In the specific case of fructose-1,6-bisphosphatase, it has been proposed that glucose induces its sequestration within double membrane vesicles, which eventually fuse with the vacuole by a process that requires protein synthesis [14].

Something similar occurs with the methylotrophic yeasts *Pichia pastoris* and *Hansenula polymorpha* when they are incubated in a medium containing methanol [15,16]. This induces the synthesis of enzymes that facilitate methanol uptake, such as alcohol oxidase in peroxisomes and formate dehydrogenase in the cytosol, as well as an overproduction of peroxisomes. When these yeasts are next cultured in a medium with glucose, formate dehydrogenase and the peroxisomes produced in excess are selectively degraded [15]. In the specific transfer from methanol to glucose, peroxisomes are degraded either by macroautophagy (macropexophagy, which sequesters single peroxisomes within pexophagosomes that fuse with the vacuole to degrade their content) in *H. polymorpha* [16] or by microautophagy (micropexophagy, which sequesters and degrades a cluster of peroxisomes within vacuolar membranes and a micropexophagy specific apparatus) in *P. pastoris* [15]. Pexophagy requires some specific proteins, such as for example Vac8 on the vacuolar membrane, the peroxisome receptor Atg30 or the peroxins Pex3 and Pex14 on the membrane of peroxisomes [17,18,19]. Other proteins involved in the activation of the selective autophagy triggered by glucose have been identified in yeast. One of those, Ras2, is at least involved in the degradation of fructose-1,6-bisphosphatase, because its mutants block the degradation of this protein [20], and other proteins, such as Gpr1 and Gpa2, are required in pexophagy for glucose sensing [21].

The data mentioned above indicate that some types of specific autophagy are induced by glucose. However, other experiments indicate that glucose could also produce in yeast the opposite effect, namely an inhibition of autophagy, at least in the case of the nonselective autophagy. For example, this can occur in *S. cerevisiae via* inhibition by glucose of the expression of Snf1p, the closest yeast homologue of AMPK (AMP-activated protein kinase). Both Snf1p and the cyclin-dependent kinase Pho85p have been described to control autophagy in opposite directions, activating and inhibiting it, respectively, *via* the autophagic proteins Atg1 and Atg13 [22]. Another example is provided by *S. cerevisiae* maintained in a medium with plenty of nutrients. Under these conditions, autophagy is inhibited via PKA (cAMP-dependent protein kinase A) and also, and independently, via TorC1 [23,24]. Both kinases phosphorylate Atg13 at different sites and inhibit, in a different way, the Atg1 complex [24], a key site that integrates different autophagic signals, as indicated also by the above reported effects of Snf1p and Pho85p [22]. In this regard, it is relevant that under glucose deprivation the regulatory and catalytic subunits form the PKA inactive complex [25], because this supports the inhibitory role of glucose on autophagy. Sch9, the closest yeast homologue of mammalian S6K1 and a substrate of TorC1, is also involved in nutrient sensing as an inhibitor of autophagy and in cooperation with PKA [26], although it is apparently less important.

In summary, while it is known that depletion of nitrogen mainly inactivates TorC1 and induces autophagy, the signalling pathways by which glucose-deprivation produces the same effect are less known. It is possible that in yeast TorC1 mainly responds to nitrogen levels while the response to carbon levels occurs mainly via the Ras/PKA pathway [27] and also, but to a lesser extent, via Snf1, TorC1 and Sch9, as described before.

In addition to the sequestration step, glucose in yeast can also affect positively autophagy at later steps, for example regulating the assembly of the V-ATPase, which, as mentioned before, consumes ATP. The V-ATPase is a supramolecular complex responsible for the acidification of organelles in eukaryotic cells [28] and has two main domains, V_1_, responsible for ATP hydrolysis, and V_o_, responsible for proton translocation. In yeast, glucose is important to modify the activity of the V-ATPase independently of protein synthesis [29]. Thus, in the absence of glucose, only 15–20% of the subunits of the V_1_ domain remain attached to V_0_, while with glucose this figure rises to 50–70% [30]. This reduces ATP consumption when the availability of glucose is limited, because the separation of the V_1_ and V_0_ portions produces the loss of their respective functions. To explain how glucose increases the V_1_–V_o_ coupling, an activation of the proton pump Pma1 at the plasma membrane that produces an increase in cytosolic pH [31] and a regulation by Ras/cAMP/PKA [32] have been postulated.

In summary, glucose in yeast can both induce and inhibit autophagy. For example, glucose induces a selective autophagy during an adaptive phase to specifically remove enzymes or organelles previously overproduced, and increases also the assembly and the activity of the V-ATPase and, thus, of the vacuolar acid hydrolases. However, glucose in a nutrient-rich medium can also suppress nonspecific autophagy to prevent an unnecessary autodigestion of the cell, and this mainly occurs via Ras/cAMP/PKA but also via TorC1 and Sch9 and by inactivation of Snf1p.

## 3. Regulation of Autophagy by Glucose in Mammalian Cells

In mammalian cells, as in yeast, autophagy is a process highly regulated by nutrients. Over the last years, the effects of amino acids on autophagy have been extensively studied. However, the role of glucose is less known, despite its implication in diseases such as diabetes or different types of cancer. In mammals low and high blood glucose levels induce the production and release of, respectively, glucagon by alpha cells and insulin by beta cells of pancreas. It is also well known that, for example in liver, glucagon activates autophagy while insulin inhibits it. However, the amount of glucose in the environment may also produce an autophagic response unrelated to these hormones. This response is mainly due to the role of glucose as an important determinant of the cellular energy status, but also to other glucose effects, such as oxidative stress and ROS accumulation. In the following sections we will not review the glucose effects on autophagy mediated by hormones, because they have been extensively described elsewhere, and we will deal exclusively with those other glucose effects.

As in yeast, in mammalian cells the availability of glucose promotes the assembly, *via* PI3K (phosphatidylinositol 3-kinase), of the V-ATPase [33] favouring the acidification of lysosomes required for the optimal activity of the lysosomal hydrolases. Although further details on this signalling pathway are unknown, AKT and p70S6K have been discarded as regulators [33]. Since lysosomal degradation is a late step in autophagy, these data could suggest a positive role for glucose in autophagy. However, only few works have postulated a positive effect of glucose on autophagy. For example, Ravikumar *et al.* [34] showed in cultured cells that glucose induces autophagy by inhibition of the main negative regulator of autophagy, mTOR. In addition, trehalose, a disaccharide composed of two molecules of glucose, activates autophagy, as demonstrated by an increased degradation of aggregates of huntingtin and α-synuclein mutants [35,36].

In contrast to these data, most investigators consider that in mammalian cells glucose inhibits autophagy [37,38,39,40]. When energy depletion occurs in cells incubated in a glucose-free medium, autophagy is activated as a mechanism to restore ATP from cellular components [41]. This drop in energy results in an increase in the AMP/ATP ratio, which will be sensed by AMPK to induce autophagy [42] by different mechanisms that will be later described. However, other authors conclude that ATP depletion is not necessary to induce autophagy by glucose deprivation. For example, endoplasmic reticulum (ER) stress caused by 2-deoxy-D-glucose (a non-metabolizable glucose analogue, 2-DG) induces autophagy [43]. Although 2-DG treatment reduces intracellular ATP content and activates AMPK, it is the restoration of the ER homeostasis (by mannose) what causes a decrease in LC3-II levels without affecting ATP or AMPK [43]. In addition, autophagy can be also stimulated by an increase in reactive oxygen species (ROS), caused both by glucose deprivation or a high glucose concentration, which can produce ER stress [44,45,46].

In the following sections, we will discuss different signalling pathways that regulate autophagy by glucose. In spite of the apparent importance of PKA in the signalling pathways by which glucose regulates autophagy in yeast, its relevance in mammalian cells remains elusive and, therefore, will not be mentioned here. Some of the implicated proteins, such as AMPK, p53 or p27^KIP1^, induce autophagy when ATP decreases in cells incubated without glucose. Others, such as p38, ERK (extracellular signal-regulated protein kinase) or JNK (c-Jun amino-terminal kinase), respond to ROS generated by an excessively low or high availability of glucose, activating autophagy. Finally, we will also mention the possible role of IKK (IκB kinase) and FOXO (forkhead class O) proteins in the induction of autophagy by glucose, which occurs without an apparent relation with ATP or ROS. All this reflects the existence in mammalian cells of a complicated network that interconnects different signalling pathways in the regulation of autophagy by glucose.

### 3.1. Role of Energy-Dependent Pathways

**Figure 2:**
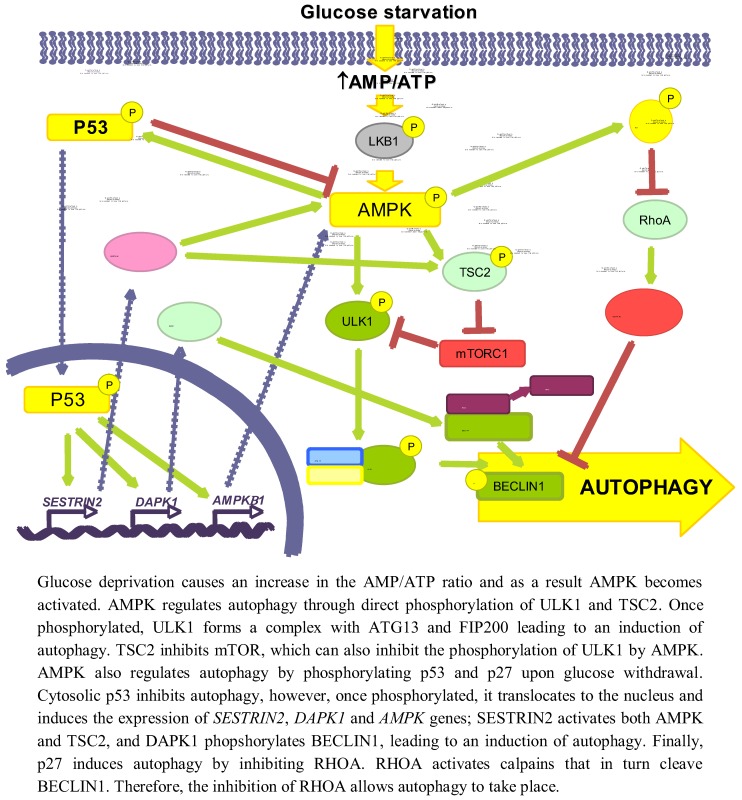
Role of energy dependent pathways.

Glucose is an important and immediate source of energy in the form of ATP. Therefore, it is logical to consider that the regulation of autophagy by glucose may be explained by changes in energetic levels. As mentioned above, AMPK is an important stimulator of autophagy that is activated by a drop in ATP levels produced, for example, by glucose withdrawal [47]. Induction of autophagy by AMPK not only is due to mTORC1 inhibition [48], but also to the activation of other signalling pathways, such as p53 [49] or p27^KIP1^ [50] (see Figure 2).

#### 3.1.1. AMPK/mTOR

AMPK collaborates in the maintenance of energy homeostasis by triggering catabolic processes that produce energy and by turning off biosynthetic processes that consume ATP [51]. AMP binds to the Crystathionine-β synthase domains of the γ subunit of AMPK [52] causing a conformational change that exposes the T172 from subunit α [53], which allows its phosphorylation by LKB1 [54].

When investigating the role of AMPK in autophagy, it is necessary to reconsider some former results in which chemical regulators of AMPK activity were used, since they can influence autophagy independently of this kinase (discussed in [55]). For example, the activator AICAR (5-aminoimidazole-4-carboxamide-1-β-D-ribofuranoside) inhibits autophagy because it blocks the interaction between PI3K-class III and BECLIN1 [56] and the inhibitor Compound C activates autophagy by suppressing the AKT-mTORC1 pathway in cancer cells [57]. Although silencing of AMPK can induce autophagy to protect pancreatic β-cells from a high (30 mM) glucose concentration [58], now it is generally accepted that AMPK positively regulates autophagy [59]. AMPK activates autophagy through TSC phosphorylation and mTORC1 inhibition [60]. In the absence of an effect of AMPK, mTORC1 is also a critical factor in the energetic equilibrium, since the inhibition of mTORC1 with rapamycin in *TSC2^-/-^*MEFs incubated in a glucose-free medium increases the ATP levels [61]. More recently, it has been shown a competition between AMPK and mTORC1 for the phosphorylation of ULK1 [42,62]. Under glucose deprivation, AMPK phosphorylates ULK1 at S317 and S777 inducing autophagy, whereas phosphorylation at S757 by mTORC1 inhibits it [42]. Furthermore, this last phosphorylation, as well as the overexpression of RHEB (a GTPase that is a TSC substrate and activates mTORC1) in *TSC^-/-^* MEFs, disrupts the AMPK/ULK1 interaction, which is restored by rapamycin [42]. Finally, under glucose deprivation the expression of the mTORC1 inhibitor DEPTOR is also induced to enhance autophagy [63,64].

Since AMPK is an energy sensor, its activity is important in many human diseases, in most cases in relationship with glucose levels and autophagy. This is highlighted for example in carcinomas in which the glycoprotein MUC1 is overexpressed. These cells incubated under low glucose conditions (1 mM) show a higher AMPK activity than normal cells [65] and, since autophagy is more active producing metabolites that generate energy, this provides them with a survival advantage.

#### 3.1.2. p53

Glucose deprivation induces the phosphorylation of p53 by AMPK in Ser15 (Ser18 in mice) [66,67,68]. Thus, in 293T cells expressing a dominant negative form of AMPK under low glucose conditions (0.5 mM), Ser18 is no longer phosphorylated [66]. In WI-38 HDF cells, glucose deprivation activates the AMPK/p53 pathway through ATM kinase and an increased expression of IFI16, leading to an induction of autophagy [49]. Notwithstanding, p53 has also been shown to be phosphorylated at the same site in *AMPK^-/- ^*MEFs incubated in glucose free medium [38], suggesting that other kinases might also be involved. This phosphorylation stabilizes p53 because it prevents its ubiquitination and subsequent degradation by proteasomes [69]. In low glucose conditions, phosphorylation of p53 results in mTOR inhibition and induction of autophagy [67], through a mechanism that involves p53 translocation to the nucleus [70], where it binds to promoter regions to induce the expression of different genes implicated in autophagy, *e.g.*, *SESTRIN 2* and *DAPK-1* (death-associated protein kinase 1). SESTRIN 2 activates AMPK and induces TSC2 phosphorylation to inhibit mTORC1 [71]. DAPK-1 phosphorylates BECLIN1 and prevents the formation of the BECLIN1/BCL-2 complex that inhibits autophagy [72]. Other genes inducing autophagy also regulated by p53 are *AMPKB1* [68], *DRAM* (damage-regulated autophagy modulator) and *TSC2* [73,74]. The fact that under glucose deprivation AMPK phosphorylates p53 and induces its nuclear translocation, and that AMPK is activated by p53 transcription targets and is itself a p53 target, suggests the existence of a positive feedback loop between AMPK and p53.

The cytoplasmic-nuclear shuttling of p53 has a major role in autophagy regulation [75]. Whereas nuclear p53 induces autophagy, it has been shown that cytosolic p53 has a negative role in autophagy that is accompanied by a reduced phosphorylation of AMPK and TSC2 and an increased activation of mTOR, as indicated by the phosphorylation of p70S6K [76].

#### 3.1.3. p27^KIP1^

Glucose deprivation increases the phosphorylation (at S83, T170 and mT197/hT198) of the cyclin-dependent kinase inhibitor p27^KIP1^ through LKB1/AMPK, allowing its stabilization [50,77]. HeLa cells, which do not express LKB1, show low levels of both total and phosphorylated p27^KIP1^, even upon glucose starvation. Reconstitution of these cells with LKB1 produces an increase in both total and P-T198 p27^KIP1 ^levels in glucose free medium, as well as in ACC phosphorylation [50]. All these results suggest that p27^KIP1^ is activated by LKB1/AMPK during glucose deprivation. Moreover, glucose deprivation induces the expression of p27^KIP1^, while moderate increments in D-(+)-glucose reduces it [78]. Conversely, there is one report showing that under glucose starvation, p27^KIP1 ^is ubiquitinated by SIAH/SIP and degraded by proteasomes [79]. This degradation of p27^KIP1 ^is associated with a decrease in cell motility, but the effect on autophagy was not studied.

Phosphorylation of p27^KIP1 ^by AMPK under glucose starvation determines, in addition to its stabilization, its cellular localization. Thus, in NIH3T3 cells expressing a constitutively active form of AMPK, only 10% of p27^ KIP1^ presents a nuclear localization, whereas in cells that express a dominant negative AMPK this percentage increases up to 70% [77]. This can be explained because phosphorylated p27^KIP1^ is sequestered in the cytosol by 14–3–3 proteins, which prevent its binding to the IMPORTINα, responsible of its transport to the nucleus [80].

p27^KIP1 ^has been shown to induce autophagy under glucose deprivation conditions, since a prolonged incubation without glucose produces an increment in the number of autophagic vacuoles in *p27^+/+^* MEFs, while autophagy is repressed in *p27^-/-^* MEFs. In addition, ectopic expression of the phosphomimic p27 T198D mutant is sufficient to induce autophagy [50].

Although the precise mechanism by which p27^KIP1^ activates autophagy is not known, it could be mediated by its effect on the RHOA-GTPase. Cytosolic p27^KIP1^ prevents the activation of RHOA-GTPase [81,82] and it is known that RHOA induces the activation of calpains involved in BECLIN1 degradation [83]. 

### 3.2. Role of Oxidative Stress-Dependent Pathways

Apart from variations in the energy levels, there are other changes that take place in the cells depending on the availability of glucose. Both glucose deprivation [84,85] and high levels of glucose alter the oxidative phosphorylation leading to mitochondrial hyperpolarization [86,87] and produce an accumulation of ROS that increases autophagy. For example, it has been shown that glucose withdrawal in cultured neonatal rat cardiac myocytes causes a two fold increase in ROS levels and a decrease in glutathione levels, which leads to the activation of autophagy [44]. More specifically, Chen *et al.* [88] established that superoxide is the main ROS responsible for autophagy induction, at least in response to glucose deprivation, and Scherz-Shouval *et al.* [89] showed that this increase in autophagy induced by ROS is mediated by regulating the activity of ATG4.

**Figure 3:**
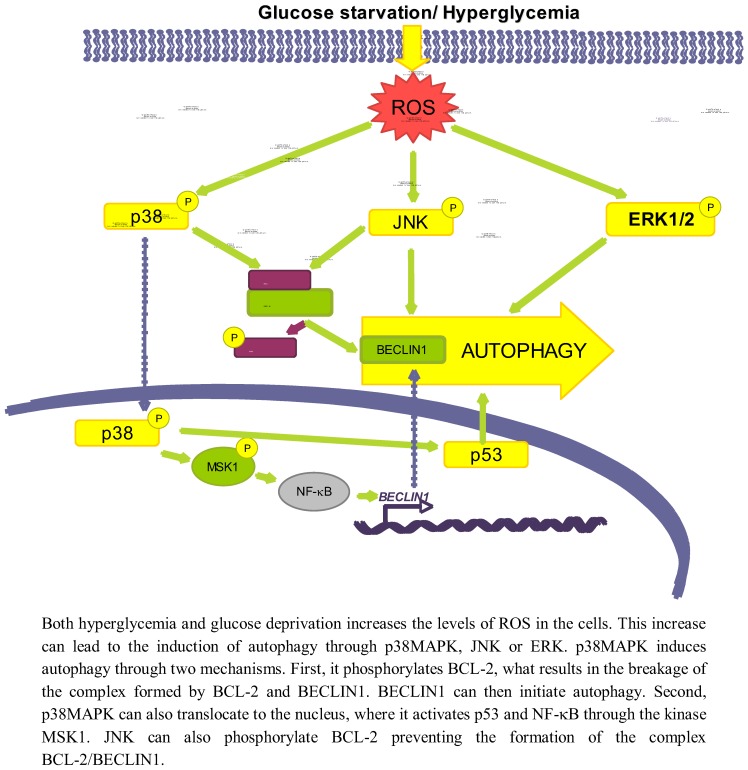
Role of oxidative stress dependent pathways.

This increase in ROS levels is subsequently sensed by proteins such as AMPK [46], p38MAPK, JNK or ERK, which will ultimately play a role in the activation of autophagy (see Figure 3). Since AMPK has been discussed previously, we will not give here further details on this kinase.

#### 3.2.1. p38MAPK

p38MAPK is activated upon oxidative stress caused both by glucose deprivation and hyperglycemia [85,90]. There are conflicting data about the role of p38 in autophagy. While it has been shown that the activation of p38MAPK by ROS or ER stress induces autophagy [91,92], this kinase has been also described to act as an autophagy inhibitor [93].

Starting with the positive effect on autophagy, it is known that once the p38MAPK is activated by different stimuli it can phosphorylate BCL-2 [94], allowing in this way the release of BECLIN1 from the BCL-2/BECLIN1 complex to initiate autophagy [95]. p38MAPK can also activate autophagy at a different level, by translocating to the nucleus to phosphorylate the downstream kinase MSK1/2, which in turn activates NF-κB, and p53 [96] likewise implicated in the positive regulation of autophagy [91]. Furthermore, data from our laboratory support a positive role for p38MAPK on autophagy induction, since we observe that addition of glucose under starvation conditions leads to p38MAPK phosphorylation and to an increase in autophagy (unpublished observations).

As for an inhibitory role, it has been described, for example, that phosphorylation of p38MAPK increases its affinity with the p38 Interacting Protein (p38IP), competing with ATG9 for its binding. As a result, there is a decrease in the formation of the p38IP/ATG9 complex, needed for autophagosome formation [93]. However, most of the studies that claim an inhibitory role for p38MAPK are based on observations using p38MAPK inhibitors such as SB203580 or SB202190 [97,98,99], and it has been recently described that these inhibitors induce the formation of autophagic vacuoles independently of p38MAPK [100,101]. Therefore, the role of p38MAPK on autophagy should be still revised, although most data favour a positive effect.

#### 3.2.2. JNK

Another effect of oxidative stress due to both a high glucose concentration or glucose deprivation is the increased expression and activation of JNK [102,103]. For example, glucose deprivation produces an increase in the cellular H_2_O_2_ levels, which ultimately will lead to the activation of the ASK1/SEK1/JNK pathway [103]. In addition, it has been established that under low glucose conditions, K-RAS induces autophagy through JNK activation [104].

A high glucose concentration leads to an interaction of JNK with other regulatory pathways such as ERK/p53 [105] or NF-κB [102], but the exact mechanism by which low glucose conditions induce autophagy via JNK has not been described. However, it is known that during nutrient deprivation JNK regulates autophagy by phosphorylating BCL-2 at residues T69, S70 and S87 and, once phosphorylated, BCL-2 dissociate from BECLIN1 allowing autophagy to take place [95]. Therefore, it is quite probable that the same occurs under glucose deprivation.

#### 3.2.3. ERK

ERK1/2 is also considered an activator of autophagy, because its inhibition lessens the induction of autophagy produced by different stimuli [106,107]. In this sense, it has been described that under amino acid deprivation ERK1/2 induces autophagy either through a trimeric G protein [108,109] or through the non-canonical module MEK/ERK downstream of AMPK and upstream of TSC and mTOR [106]. However, Corcelle *et al.* [110] have also shown that a sustained activation of ERK by the carcinogen Lindane has the opposite effect since it inhibits the maturation of autophagosomes.

Since it is known that glucose induces both ROS and AMPK, which activate ERK1/2 [106,111], it seems possible that ERK1/2 is one of the molecules involved in activation of autophagy by glucose. In fact, ERK1/2 is phosphorylated under high glucose concentrations [112,113]. A more direct relationship among glucose availability, ERK activation and autophagy was provided by Yan *et al.* [105] that showed that in diabetic GK rats, hyperglycemia-associated oxidative stress may induce autophagy through upregulation of the ROS-ERK/JNK-p53 pathway.

### 3.3. Role of Other Pathways

**Figure 4:**
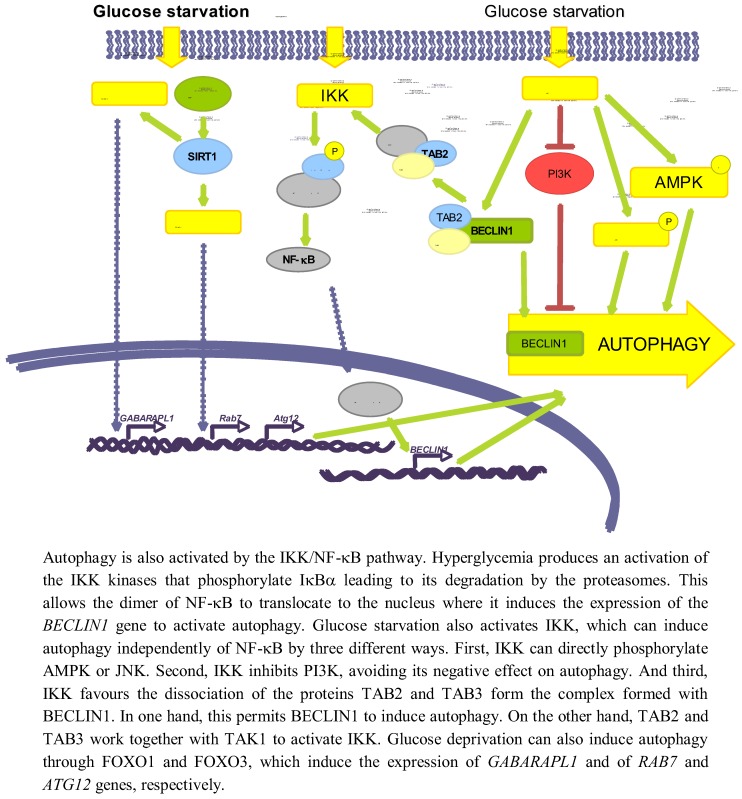
Other pathways involved in autophagy regulation by glucose.

In addition to the signalling pathways related with energy changes and ROS induction that appear to be implicated in the regulation of autophagy in response to glucose levels, there are others implicated in this regulation. Here we will describe the role of NF-κB, SIRT1 and FOXO (see Figure 4).

#### 3.3.1. NF-κB

Several studies have established an effect of a high glucose concentration in the activation of IKK [114], which can be also produced by deprivation of amino acids and serum [115]. Hyperglycemia activates IKK, leading to IκBα phosphorylation and degradation, and subsequent activation of NF-κB trough a mechanism that involves PKC [116]. It has been also shown that a high glucose concentration produces an activation of NF-κB coupled with an induced translocation to the nucleus of the NF-κB heterodimer p65/p50 [117].

Independently of the above-mentioned reports, it has been shown a positive correlation in the activities of NF-κB and autophagy. Thus, Copetti *et al*. [118] described that activation of T cells with PMA/ionomycin leads to p65-induced expression of BECLIN1. Comb *et al.* [119] recently showed that under starvation conditions, expression of BECLIN1, LC3 and ATG5 relay only on IKK complex but not on NF-κB. It has been also described that IKK can directly induce autophagy through the phosphorylation of JNK1 and AMPK [120]. Finally, starvation produces the dissociation of the complex formed by TAK1-binding proteins 2 and 3 (TAB2 and TAB3) and BECLIN1. In one hand, this allows BECLIN1 to induce autophagy, and on the other hand TAB2/TAB3 activates TAK1 that in turn activates IKK [121]. IKK can also phosphorylate the p85 regulator subunit of PI3K at Ser690, inhibiting the PI3K/AKT pathway and thus suppressing its inhibitory role on autophagy [115].

Although the direct relationship among glucose availability, induction of NF-κB and a subsequent effect on autophagy has not been described yet, these data suggest a possible role of NF-κB on glucose-induced autophagy. Therefore, more studies are needed to verify this possibility.

#### 3.3.2. SIRT1 and FOXO

SIRT1 is a NAD^+^-dependent deacetylase that has been reported to deacetylate, upon nutrient deprivation (HBBS medium), ATG5, ATG7 and ATG8 proteins to induce autophagy [122]. Under serum and glucose deprivation the expression of SIRT1 is induced, that in turn deacetylates FOXO1 and FOXO3 [39,123]. As a consequence, FOXO proteins translocate to the nucleus where they induce the expression of *RAB7* (a GTPase protein implicated in the fusion of autophagosomes with endosomes and lysosomes) [39], *GABARAPL1*, *LC3* and *ATG12* (which participates in the elongation of the autophagic vesicles) genes [123]. Therefore, glucose deprivation can induce autophagy *via* SIRT1/FOXO1/3.

## 4. Pathological Implications of Autophagy Regulation by Glucose

In addition to its obvious importance for a better understanding of the physiological roles of autophagy, the issue of the regulation of autophagy by the available glucose levels has also important pathological implications. We will present here only two examples in relationship with two important pathologies: cancer and diabetes.

The induction of autophagy under conditions of glucose deprivation is very important for the maintenance of cell homeostasis. When the availability of glucose is a limiting factor and thus depletion of energy occurs, as is the case in most cancers, an increased autophagy allows to obtain energy from the superfluous cell constituents. Cancer cells survive and even grow in poor nutrient microenvironments thanks to an induction of autophagy via AMPK, p53 or JNK [124]. In the case of K-RAS transformed cells, JNK induces autophagy and when these cells are incubated under low glucose conditions their growth is not affected [104]. In addition, human hepatoma tissues with low glucose uptake and high K-RAS expression show increased LC3-II levels [104]. Thus, induction of autophagy in environments with low glucose availability seems to be essential to maintain the survival of tumour cells.

We have also discussed above that glucose deprivation, as well as hyperglycemia, induces autophagy by signalling pathways mediated by ROS production. This is of great importance in the pathology of diabetes, where the production of ROS by hyperglycemia has been shown to be an important factor influencing its clinical complications [125]. The hyperglycemic environment in which the cells survive leads to ROS accumulation, mitochondrial dysfunction and ER stress that causes an incorrect folding of the proteins, which as a consequence tend to form aggregates [126]. Therefore, in pancreatic β-cells an increase in autophagy takes place in order to degrade these aggregates that cannot be degraded by proteasomes. In this sense, autophagy plays a protective role [127], favouring the structure maintenance, mass and function of β-cells [128]. For example, it has been reported that incubation for 72 h of pancreatic cells in the presence of 30 mM glucose induces autophagy as a survival mechanism and that inhibition of this autophagy with siRNAs targeting ATG7 produces an increased activity of CASPASE 3 that compromises the survival of β-cells [58]. Along the same line, a deficiency in autophagy during hyperglycemia will produce an increase in oxidative stress, causing the formation of atherosclerotic plaques [129]. Although autophagy in hyperglycemia is mainly protective, it has been also shown that an excess can also have negative consequences by diminishing mitochondrial mass, as it occurs in non-obese diabetic G-K rats where autophagy inhibition can lead to a reduction in oxidative stress, allowing the recovery of the mitochondrial function [105]. The ER stress caused under hyperglycemic conditons, and also under high fat diet or obesity, causes a sustained activation of JNK1, which has been implicated in the development of obesity and insulin resistance [130,131]. In fact, treatment of mice with JNK inhibitors reduces hyperglycemia and improves insulin sensitivity [132,133]. A direct relationship among diabetes, JNK1 activation and autophagy has been shown in G-K rats [105].

Diabetic patients are at significantly higher risk of ischemia/reperfusion injury [134]. During ischemia, there is a constraint of blood supply, causing a deficiency in the oxygen and glucose provided to the cells for its cellular metabolism. After ischemia, reperfusion must occur to allow the survival of the tissue. In cardiomyocytes, glucose deprivation during ischemia produces an increase in autophagy dependent on AMPK, RHEB/mTORC1 and SIRT1/FOXO3 [48,135]. Presence of oxygen during this step is of main importance for autophagy to take place, as its absence impairs autophagy [136]. This increased autophagy confers a protective role, leading to survival of the cells under these conditions [48,135]. The same has been also shown *in vivo* during ischemia/reperfusion [137,138]. Treatment with an mTOR inhibitor to induce autophagy after myocardial infarction decreases the infarct size [137]. In the same line, during reperfusion, autolysosome maturation is inhibited [136]. This impairment of late steps in autophagy, associated with increased ROS, causes cardiomyocyte death [138]. However, it has been also shown that an excessive autophagy later during reperfusion can cause extensive cell death [48].

## 5. Concluding Remarks

Although some of the signalling pathways that orchestrate in yeast the regulation of autophagy by glucose have been conserved in mammalian cells, a more complex signalling network appears to be involved in these cells. Here, AMPK, which is activated by a deficit in energy, is the most important kinase of these signalling pathways. This kinase exerts its effect mainly, but not exclusively, through the mTOR kinase, the main nutrient sensor in the cells. However, there are other proteins that are also implicated in the regulation of autophagy downstream of AMPK in the response to glucose deprivation, *e.g.*,p53 and p27^KIP1^, also activated by ATP depletion. In addition, ROS also influence autophagy in response to glucose deprivation or hyperglycemia, by signalling pathways that implicate other kinases such as p38MAPK, JNK or ERK1/2. Finally, glucose-induced autophagy can be also regulated by IKK/NF-κB or FOXO, independently of ROS or the ATP levels and AMPK function.

The importance of the regulation of autophagy by glucose is highlighted by human diseases in which glucose levels are affected. For example, autophagy serves to preserve cell integrity in cancer cells growing in glucose-limiting environments and in pancreatic β-cells under diabetes. Therefore, understanding the mechanisms of autophagy regulation by glucose is of major interest in order to better understand these and other pathologies.
